# Ligustrazin increases lung cell autophagy and ameliorates paraquat-induced pulmonary fibrosis by inhibiting PI3K/Akt/mTOR and hedgehog signalling via increasing miR-193a expression

**DOI:** 10.1186/s12890-019-0799-5

**Published:** 2019-02-11

**Authors:** Ming-wei Liu, Mei-xian Su, Deng-yun Tang, Li Hao, Xiang-Han Xun, Yun-qiao Huang

**Affiliations:** 1 Department of Emergency, the First Affiliated Hospital of Kunming Medical University, 295 Xichang Road, Kunming, 650032 Yunan People’s Republic of China; 2 Emergency Intensive Care Unit, the Second Affiliated Hospital of Kunming Medical University, Kunming, 650106 China; 3 Skin Disease Prevention Institute of Wenshan Zhuang and Miao autonomous prefecture, Wenshan, 655500 China; 4 Intensive Care Unit, the Yan-an Affiliated Hospital of Kunming Medical University, Kunming, 650106 China; 5 Department of Thoracic Surgery, People’s Hospital of Fuyuan County, Qujing, 655500 China

**Keywords:** Lung fibrosis, Ligustrazin, Paraquat, miR-193a, Akt, mTOR, Oxidative stress, Mice

## Abstract

**Background:**

Reactive oxygen species (ROS) levels largely determine pulmonary fibrosis. Antioxidants have been found to ameliorate lung fibrosis after long-term paraquat (PQ) exposure. The effects of antioxidants, however, on the signalling pathways involved in PQ-induced lung fibrosis have not yet been investigated sufficiently. Here, we examined the impacts of ligustrazin on lung fibrosis, in particular ROS-related autophagy and pro-fibrotic signalling pathways, using a murine model of PQ-induced lung fibrosis.

**Methods:**

We explored the effects of microRNA-193 (miR-193a) on Hedgehog (Hh) and PI3K/Akt/mTOR signalling and oxidative stress in lung tissues. Levels of miR-193a, protein kinase B (Akt), phosphoinositide 3-Kinase (PI3K), ceclin1, mammalian target of rapamycin (mTOR), sonic hedgehog (SHH), myosin-like Bcl2 interacting protein (LC3), smoothened (Smo), and glioma-associated oncogene-1 (Gli-1) mRNAs were determined with quantitative real-time PCR. Protein levels of PI3K, p-mTOR, p-Akt, SHH, beclin1, gGli-1, LC3, smo, transforming growth factor-β1 (TGF-β1), mothers against DPP homologue-2 (Smad2), connective tissue growth factor (CTGF), collagen I, collagen III, α-smooth muscle actin (α-SMA) nuclear factor erythroid 2p45-related factor-2 (Nrf2), and p-Smad2 were detected by western blotting. In addition, α-SMA, malondialdehyde, ROS, superoxide dismutase (SOD), oxidised and reduced glutathione, hydroxyproline, and overall collagen levels were identified in lung tissues using immunohistochemistry.

**Results:**

Long-term PQ exposure blocked miR-193a expression, reduced PI3K/Akt/mTOR signalling, increased oxidative stress, inhibited autophagy, increased Hh signalling, and facilitated the formation of pulmonary fibrosis. Ligustrazin blocked PI3K/Akt/mTOR and Hh signalling as well as reduced oxidative stress via increasing miR-193a expression and autophagy, all of which reduced pulmonary fibrosis. These effects of ligustrazin were accompanied by reduced TGF-β1, CTGF, and Collagen I and III expression.

**Conclusions:**

Ligustrazin blocked PQ-induced PI3K/Akt/mTOR and Hh signalling by increasing miR-193a expression, thereby attenuating PQ-induced lung fibrosis.

## Background

A large number of the fatalities caused by paraquat (PQ) are related to its toxicity and the lack of effective treatments [1]. The guidelines for treating patients with PQ poisoning have not been fully developed, but current treatments include single supportive care and combinations of haemodialysis, antioxidant therapy, immune-modulation, and haemoperfusion [2]. Despite these treatments, the current mortality rate for PQ poisoning is still > 50% [3]. Oxidative stress is an important molecular mechanism of PQ-induced pulmonary fibrosis [4] that often occurs in the lung after PQ exposure, disturbing the balance between oxides and peroxides and increasing reactive oxygen species (ROS) levels [5]. ROS induce pulmonary fibrosis by promoting lung cell apoptosis and reducing autophagy levels in alveolar epithelial cells [6].

Ligustrazin is extracted from the roots and stems of *Ligusticum chuanxiong* Hort (Chuan Xiong) and can scavenge ROS, regulate nitric oxide production and prevent peroxynitrite formation [7]. Ligustrazin scavenges oxygen free radicals and affects cell toxicity [8]. Li et al. suggested that the cardioprotective mechanism of ligustrazin involved blocking free radical formation and lipid peroxidation [9]. Wang et al. reported that ligustrazin protected the myocardium by activating Superoxide Dismutase (SOD) and Glutathione Peroxidase (GSH-Px), in addition to stimulating Heat-shock Protein-70 (HSP70) mRNA and protein expression [10]. Previous studies have made the PQ model one of the best characterized models of fibrosis, as this method invokes a highly reproducible oxidative stress response that leads to fibroblast proliferation, collagen deposition, and ultimately incurable pulmonary fibrosis [11]. In this evaluation, we utilized a PQ-induced pulmonary fibrosis model to analyze the mechanism of ligustrazin against pulmonary fibrosis.

In many biological processes, microRNAs (miRNAs) are main regulators of gene expression [12] and many disease-related miRNAs have been reported recently [13–15]. Additionally, studies of miRNAs in autophagy and apoptosis have shown their functional effects using in vivo models [15–18]. However, the precise functions of miRNAs in fibrotic diseases, especially lung fibrosis, are unknown. Autophagy is involved in the pathogenesis of pulmonary diseases [19]. In macro-autophagy, a double-layer membrane forms around an autophagosome. Autophagy levels are decreased in lung tissues of idiopathic pulmonary fibrosis patients [20], and immunohistochemistry has indicated altered p62 expression in idiopathic pulmonary fibrosis lung tissues, suggesting reduced autophagic activity [21]. Additionally, the autophagy-associated protein Beclin1 was decreased in idiopathic pulmonary fibrosis fibroblasts [12]. Mammalian Target of Rapamycin (mTOR) is a serine/threonine kinase [22], and mTOR-dependent signalling regulates autophagy. Autophagy can be inhibited by activating the Protein Kinase B (AKT)/mTOR pathway, whereas loss of signalling through this pathway leads to the loss of mTOR repression [23].

Reactivation of Hedgehog (Hh) signalling has been implicated in fibrosis of various organs [24]. Both non-alcoholic steatohepatitis and chronic cholestasis have been characterized by increased Hh signalling in fibrosis. Hh signalling activates hepatic stellate cells to develop the myofibroblastic phenotype [25]. In the majority of adult tissues, Hh signalling is not stimulated. Nevertheless, evaluations have recently indicated that Hh signalling can be reactivated during fibrosis or tissue remodelling [26–28]. Reactivation of Hh signalling has been demonstrated to happen in the lungs of patients with idiopathic pulmonary fibrosis [28], the fibrotic skin of scleroderma patients [30], animals with hyperoxic lung injury [29], animal models of liver fibrosis [32, 33] and human non-alcoholic fatty liver [31]. Sonic Hedgehog (SHH) ligand is upregulated in airway epithelial cells in lung fibrosis and Patched1 (Ptch1) expression is elevated in pulmonary interstitial cells [34].

Here, we studied the antioxidant ligustrazin and evaluated whether it blocked pulmonary fibrosis and we analyzed the latent signalling pathways associated with its anti-fibrotic impacts with a murine model of long-term PQ exposure. Our data show that ligustrazin ameliorated lung fibrosis and blocked ROS-dependent miR-193a activation by inhibiting Hh signalling and stimulating pro-autophagy pathways.

## Methods

### Reagents

We purchased PQ in an aqueous solution (active ingredient content: 200 g/L) from Chuandong Agrochemical Co., Ltd. (Chongqing, China). Ligustrazin was acquired from Sigma-Aldrich (St. Louis, MO, USA). The Western Lightning-Enhanced Chemiluminescence Kit was bought from Perkin Elmer (Waltham, MA, USA). TRIzol reagent came from Invitrogen (Carlsbad, CA, USA) and the SYBR Green Fluorescence Quantitative Reverse Transcription PCR (qRT-PCR) kit was purchased from Takara (Kyoto, Japan). Murine MMP-9, TGF-β1, and VEGF ELISA kits were purchased from Bender Medsystem (Vienna, Austria).

### Animals

Six-week-old female C57BL/6 mice were acquired from the Laboratory Animal Research Centre of Kunming Medical University (Kunming, China). The mice were kept under a 12-h light/dark cycle under a constant temperature (22 °C) and humidity. The mice had access to water and chow ad libitum throughout the experimental protocols. The Institutional Animal Care and Use Committee of Yunnan University approved each of the experimental procedures.

As reported in previous studies [35, 36], mice were weighed and then placed into one of four groups (*n* = 8 per group) to evaluate the protective impacts of ligustrazin on pulmonary fibrosis. PQ (10 mg/kg) or saline was intraperitoneally injected into the mice to induce pulmonary fibrosis [2]. Group 1 was untreated and treated as a control group. Group 2 was treated with ligustrazin (30 mg/kg once per day) with a tail vein injection and this group acted as the control treatment group. Group 3 were given PQ (10 mg/kg) to prompt pulmonary fibrosis and this group acted as the model group (PQ group). Group 4 consisted of eight mice that received PQ to prompt pulmonary fibrosis. Mice in Group 4 were then administered ligustrazin (30 mg/kg once per day) with a tail vein injection and was considered the treatment group (PQ treatment group). The animals were killed by cervical dislocation and anaesthetized with Zoletil 50 (20 mg/kg) that was administered intraperitoneally based on the company’s directions (Virbac, Carros Cedex, France) at 14 days following PQ injection. Mice Subsequently, the lungs were removed. A small section of the lung was stained with haematoxylin-eosin (H&E), which involved fixation with 10% formalin, paraffin embedding, sectioning, and staining.

### Cell culture

We used the methodology previously described by Wei [37]. Human lung epithelial cells (A549) (Heilongjiang Cancer Institute, Harbin, China) were grown in DMEM supplemented with 100 U/mL penicillin, 100 U/mL streptomycin, 5% foetal bovine serum, and 50 μg/L amphotericin B in a humidified atmosphere containing 5% CO_2_ at 37 °C. The cells were sub-cultured in six-well plates and kept until sub-confluence.

### Transfecting miRNA mimics, expression plasmids, and inhibitors

A549 cells were sub-cultured in six-well plates to 40% confluence. miR-193a mimics, miR-193a inhibitor, miR-193a mimic-negative control (NC), and miR-193a inhibitor-NC were combined with lipofectamine 2000 (Invitrogen). The combination was placed into the medium. After 24 h of transfection, RNA and total protein were prepared from the cells for qRT-PCR and western blotting analyses, respectively.

### GFP-LC3 imaging

A549 cells were transfected with GFP-LC3 plasmids, miR-193a mimics, inhibitors, or scrambled sequences to assay autophagy. Cells were grown on glass coverslips, fixed with 4% sucrose and 4% paraformaldehyde. They were then permeabilized for 10 min with 0.1% Triton X-100. The cells were then rinsed, mounted on cover glasses with 4,6-diamidino-2-phenylindole (DAPI; Invitrogen) and Prolong Gold anti-fade reagent. They were then visualised with an Olympus IX 81 microscope (Olympus, Tokyo, Japan). The percentage of cells with punctate GFP-LC3 fluorescence was determined by quantifying the amount of cells with punctate GFP-LC3 expression among all GFP-positive cells to quantify autophagy. At least 300 cells were scored from three arbitrarily chosen fields per condition for each experiment.

### Intravenous tail administration of AdCMV-miR-193a

A constitutively active miR-193a expression construct was injected in the mice through intravenous tail administration of 1 × 10^9^ pfu AdCMV-miR-193a. This followed the intraperitoneal administration of PQ by 14 d. Control mice received an empty adenoviral vector on the same schedule.

### MiR-193a target gene prediction and dual luciferase reporter assay

TargetScan (http://www.targetscan.org), PicTar (www.pictar.org), and miRBase (www.mirbase.org) were used to predict candidate miR-193a targets. Pancreatic acinar cells (1 × 10^5^) were cultured in 24-well plates. The cells were then transfected with SHH-3′UTR-wild-type (wt) or SHH-3′-untranslated region (UTR)-mutant (mt), and miR-193a or miR-193a-NC with lipofectamine 2000 (Invitrogen). Luciferase activity was measured at 24 h after transfection with the Dual Luciferase Reporter Assay System (Promega, Madison, WI, USA). The results were normalized to Renilla activity.

### qRT-PCR analysis

miRNAs were purified with the mirVana miRNA Isolation Kit (Ambion, Carlsbad, CA, USA) to measure miR-193a expression. miRNAs were then transcribed into cDNAs, which were amplified by PCR using Taqman primers (miR-193a, ID: 2299) to analyze the miRNAs. Pri-miRNAs were purified with TRIzol (Invitrogen) and then transcribed into cDNAs. PCR with the primers listed in Table 1 was then performed. An Applied Biosystems 7900 Fast Real-Time PCR System (Foster City, CA, USA) was used for qRT-PCR. The input amounts were normalised to U6 (Taqman probe ID: 1973), which was employed as the endogenous control gene. Expression levels were normalized to the endogenous controls. The fold changes in expression levels were determined with the 2^−∆∆Ct^ method.

**Table 1:** Primer sequences for qRT-PCR

U6	F-5’-GTCGTATCCAGTGCAGGGTCCGAGGT-3’	174 bp
	R-5’-ATTCGCACTGGATACGACCAGACTC-3’	
miR-193a	F-5’-CTCGCTTCGGCAGCACA − 3’	158 bp
	R-5’-AACGCTTCACGAATTTGCGT − 3’	
Akt mRNA	F-5’-TCACCTCTGAGACCGACACC-3’	121 bp
	R-5’-ACTGGCTGAGTAGGAGAACTGG-3’	
Beclin1 mRNA	F-5’-ATGCAGGTGAGCTTCGTGTG-3’	247 bp
	R-5’-CTGGGCTGTGGTAAGTAATGGA-3’	
LC3-IImRNA	F-5’-AAACGCATTTGCCATCACA-3’	193 bp
	R-5’-GGACCTTCAGCAGTTTACAGTCAG-3’	
mTOR mRNA	F-5’-AGAAACTGCACGTCAGCACCA-3’	217 bp
	R-5’-CCATTCCAGCCAGTCATCTTTG-3’	
SHH mRNA	F-5’-CCAATTACAACCCCGACATC-3’	178 bp
	R-5’-GCATTTAACTTGTCTTTGCACCT-3’	
Smo mRNA	F-5’-GGCTGCTGAGTGAGAAG-3’	194 bp
	R-5’-CTGGTTGAAGAAGTCGTAGAAG-3’	
Gli-1 mRNA	F-5’-CCAAGCCAACTTTATGTCAGGG-3’	215 bp
	R-5’-AGCCCGCTTCTTTGTTAATTTGA-3’	
TGF-β1mRNA	F-5′- TACCAGAAATACAGCAACA-3′	138 bp
	R-5’-TGACATCAAAAGATAACCA-3’	
Collagen I mRNA	F-5′- TCTAGACATGTTCAGCTTTGTGGAC-3′	252 bp
	R-5′- TCTGTACGCAGGTGATTGGTG-3’	
β-actin	F-5’-TACAACCTTCTTGCAGCTCC-3’	192 bp
	R-5’-ATCTTCATGAGGTAGTCTGTC-3’	

To measure mTOR, PI3K, Beclin1, Akt, LC3, SHH, Smo, and Gli-1 mRNA expression levels, total RNA was removed from frozen lung tissues (left lungs) with TRIzol reagent (Invitrogen). A single-step PCR kit (Promega) was then used for amplification. A PTC-200 DNA Engine PCR cycler (MJ Research, Inc., Hercules, CA, USA) was used for RT-PCR. Primers were designed according to published sequences of the genes and were synthesised by Invitrogen (Table 1). The loading control was β-actin. The reaction was started at 25 °C and lasted for 5 min. This was followed by annealing at 50 °C and then elongation at 70 °C. cDNA products were then diluted in RNase-free water and DNase-free water to a total volume of 250 μL. This was frozen at − 20 °C to be used for gene expression assays. Then, 5 μL of diluted cDNA, 4 μL of water, 10 μL of 1× PCR master mix, and 1 μL of the probe were mixed for every reaction. Agarose gel electrophoresis was performed and results were visualised with Gelview (Bioteke Co., Beijing, China). Semi-quantitative densitometric evaluation was conducted with Bio-Rad Universal Hood and Quantity One software (Bio-Rad, Hercules, CA, USA). mRNA expression profiles were obtained with β-actin in place as an endogenous control. The fold-change in expression levels were determined with the 2^−∆∆Ct^ method.

### Western blot analysis

Lung homogenates were prepared in a lysis buffer with 0.5% sodium deoxycholate, 50 mM Tris-HCl, 2 mM EDTA, 2 mM NaF, 1% NP-40, 0.150 mM NaCl, 1% SDS and a 1× protease inhibitor cocktail tablet (Roche, Basel, Switzerland). Protein concentrations were quantified with a BCA assay. Identical portions of protein (30 mg) from every group were loaded onto 12% tris-glycine polyacrylamide gels. Samples and pre-stained markers (Bio-Rad) were electrophoresed and then moved onto polyvinylidene difluoride membranes (Millipore, Marlborough, MA, USA). Membranes were halted for 1 h at room temperature with 5% bovine serum albumin. Membranes were then incubated at 4 °C overnight in Tris-buffered saline with Tween-20 (TBST). These primary antibodies were used: anti-PI3K (1:1000), anti-Akt (1:1500), anti-p-mTOR(1:1000), anti-p-Akt (1:400), anti-LC3-I (1:400), anti-LC3-II (1:400), anti-Beclin1 (1:1000), anti-SHH (1:400), anti-Gli-1 (1:400), anti-TGF-β1 (1:1000), anti-Smo (1:1000), anti-Smad2 (1:1500), anti-p-Smad2 (1:1500), anti-Collagen III (1:1000), anti-CTGF (1:1000), anti-Collagen I (1:1000), anti-α-SMA (1:400), anti-Nrf2 (1:1000), anti-VEGF (1:400) (all from Cell Signalling Technology, Danvers, MA, USA). Anti-CTGF I (1:1000) and anti-β-actin (1:1500) (both from Santa Cruz Biotechnology, Dallas, TX, USA) were also used. β-actin was utilized as the loading control and it was also used to verify that the identical portion of protein was loaded each time. Membranes were incubated with horseradish peroxidase-linked anti-rabbit antibodies (Pierce, Rockford, IL, USA) that were diluted 1:20000 in TBST at room temperature for 1 h. After rinsing with TBST, immunoreactive bands were viewed with enhanced chemiluminescence. Bands were determined by densitometry with Universal Hood and Quantity One software (Bio-Rad). All of the outcomes were normalized to the β-actin levels.

### Enzyme-linked immunosorbent assay (ELISA)

Bronchoalveolar lavage fluid was acquired from them animals beneath anaesthesia with 1 mL of sterile isotonic saline after 24 h following the final challenge. Lavage was implemented four times on each mouse and the total volume was measured. Lavage fluid samples were directly centrifuged at room temperature (10 min at 2000×g). Samples were then kept at − 80 °C. TGF-β1, MMP-9, and VEGF levels were measured with TGF-β1, MMP-9, and VEGF ELISA kits, respectively, based on the manufacturer’s directions. TGF-β1, MMP-9 and VEGF protein levels in each sample are expressed as mean ± standard error.

### Immunohistochemistry

Paraffin-embedded tissues were processed with the avidin-biotin immuno-peroxidase method to measure Beclin1 and α-SMA expression levels in the lungs. Antibodies against Beclin1 and α-SMA (1:100; Abcam, Cambridge, UK) were used for immunohistochemistry. All sections were evaluated independently and scored by two investigators blind to the protocol. Five high-power fields (200×) were arbitrarily chosen from every slide after staining. Positive staining in every field was counted with a true colour multi-functional cell imaging analysis management system (Image-Pro Plus, Media Cybernetics, Rockville, MD, USA). Results were expressed as positive units.

### In situ hybridisation (ISH)

To observe miR-193a expression in lung tissues, we designed a 5′-digoxigenin-labeled oligonucleotide probe to hybridise with miR-193a in situ using the MicroRNA ISH Buffer and Controls Kit (Exiqon, Woburn, MA, USA).

### Measuring intracellular ROS

We used the methodology previously described by Liu [38], quantified ROS. Cells were incubated at room temperature for 10 min with PBS with 3.3 μM 2′,7′-dichlorofluorescein (DCF) diacetate (Molecular Probes, Eugene, OR, USA) to label and measure intracellular ROS. A fluorescence-activated analysis to sort cells was performed with 1 × 10^4^ DCF-stained cells.

### Measuring malondialdehyde (MDA)

As previously described by Liu [38]. Lung tissue homogenates were obtained from the experimental groups and controls with 0.1 M Tris-HCl buffer (pH 7.4) at 4 °C. The tissue homogenates were used for biochemical measurements. MDA contents were identified colorimetrically with commercially available kits (Jiancheng Bioengineering Institute, Nanjing, China). Simply, the MDA content of the serum was quantified with the thiobarbituric acid method. In this method, a red complex formed when MDA reacted with thiobarbituric acid. Absorbance at 532 nm was quantified.

### Measuring GSH in lung tissues

We used the methodology previously described by Liu [38], Lung tissues were homogenized in ice-cold lysis buffer (10 mL, 50 mM phosphate buffer with 1 mM EDTA per gram of tissue). After centrifugation at 4 °C for 15 min (10,000×*g*), supernatants were removed. The specimens were deproteinated and then stored at − 20 °C for subsequent analyses. A GSH Assay Kit (Cayman Chemical Company, Ann Arbor, MI, USA) was used to quantify overall GSH levels.

### Hydroxyproline (HYP) assay

As previously described by Liu [38]. HYP levels were measured to determine the collagen content. In short, 10 mg of lung tissue were minced and place in 1 mL of 6 mol/L HCl. They were then hydrolysed and incubated overnight at 120 °C. Next, citric/acetate buffer was added. The pH was altered to 6.0–6.5 with 0.2 mol/L NaOH. The combination was incubated at room temperature for 20 min after 1 mL of chloramine T solution (0.05 mol/L) was added. Then, the combination was incubated for 15 min at 60 °C after adding 1 mL aldehyde perchloric acid. Then, absorbance at 550 nm was documented for each sample. The outcomes were described in micrograms per milligram of wet lung weight based on the HYP standard curve.

### Histopathology

According to previous reports [39, 40], the middle lobes of right lung sections were placed in paraffin and then fixed in 10% buffered formalin. They were then processed into 4-mm sections for Masson’s trichrome staining. The Ashcroft scoring method was used for histopathological evaluations of pulmonary fibrosis. Briefly, the lung fibrosis grades were scored on a scale of 0–8 with the following criteria: Grade 0, normal lung; Grades 1–2, minimal fibrous thickening of the alveolar or bronchiolar walls; Grades 3–4, moderate thickening of the walls without obvious damage to the lung architecture; Grades 5–6, increased fibrosis with definite damage to the lung structure; and Grades 7–8, severe distortion of the lung structure and large fibrous areas [12]. After examining 30 arbitrarily chosen regions from every specimen (magnification, 100×), the mean score from each of the regions was calculated and used as the overall fibrosis score. Scoring was performed by investigators who were blind to the protocol. Meanwhile, some specimens were frozen in 2-methylbutane and 6-μm sections were subjected to H&E staining for light microscopy assessment.

### Electron microscopy

We used the methodology previously described by Abassi [41, 42]. Lung tissues from the various experimental groups were set in 3.5% glutaraldehyde and then cleansed in 0.1 M sodium cacodylate buffer (pH 7.4). Tissue blocks of 1 mm^3^ were post-fixed for 1 h in 2% OsO_4_ in 0.2 M cacodylate buffer. They were then rinsed with cacodylate buffer to eliminate extra osmium and immersed in saturated aqueous uranyl acetate. Then they were dehydrated in a graded alcohol series, immersed again in propylene oxide, and embedded in Epon 812. Ultrathin sections (80 nm) were placed on a 300-mesh, thin-bar copper grid. They were then counterstained with saturated lead citrate and uranyl acetate. Stained sections were observed with transmission electron microscopy (Jeol 1011 JEM, Tokyo, Japan).

### Statistical analysis

All experimental data are expressed as the mean ± standard deviation. A one-way ANOVA test and Student–Newman–Keuls test were performed to contrast multiple groups. Significance was set as a *p*-value < 0.05. SPSS 13.0 (SPSS Inc., Chicago, IL, USA) was utilized for all of the statistical analyses.

## Results

### miR-193a decreased p-Akt, Beclin1, LC3-II, and autophagy levels in A549 cells

**Fig. 1:**
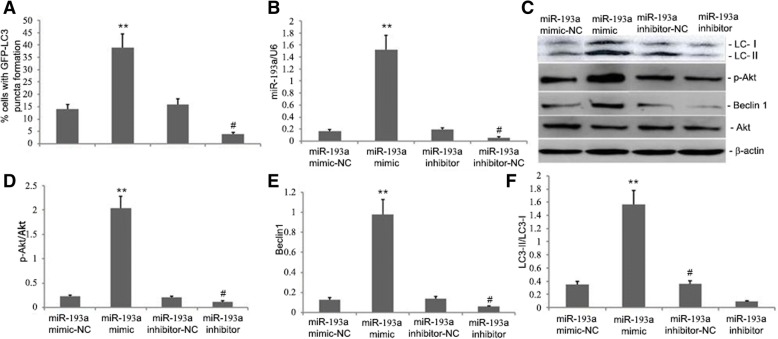
Effects of miR-193a on Beclin1, p-Akt, and LC3-II levels and autophagy in A549 cells. **a** Quantification of the percentage of A549 cells with GFP-LC3 puncta in cells that were transfected with miR-193a mimic, inhibitor or scrambled sequences. **b** MiR-193a levels in A549 cells that were transfected with miR-193a mimic, inhibitor or scrambled sequences. **c-f** Representative statistical analyses and graphs of mTOR, Akt, p-Akt, p-mTOR, Beclin1 and LC3-II levels by western blotting in A549 cells that were transfected with miR-193a mimic, inhibitor or scrambled sequences. Data that were obtained from quantitative densitometry are presented as the mean ± SEM of three independent experiments. ^*^p < 0.05 vs. controls and treatment groups. ^#^*p* < 0.05 vs. controls

To investigate the impacts of miR-193a on p-Akt, Beclin1 and LC3-II in A549 cells, miR-193a mimic, miR-193a mimic-NC, miR-193a inhibitor, and miR-193a inhibitor-NC were transfected into the cells. Then, 24-h after transfection, miR-193a levels were determined by qRT-PCR, and Akt, p-Akt, Beclin1 and LC3-II levels were assayed by western blot. MiR-193a mimic significantly elevated miR-193a levels, while miR-193a inhibitor significantly lowered miR-193a levels in A549 cells (Fig. 1b). The decrease in miR-193a decreased p-Akt, Beclin1 and LC3-II levels, thereby decreasing autophagy in A549 cells (Fig. 1a, c-f). Conversely, increased miR-193a levels significantly increased p-Akt, Beclin1 and LC3-II levels, thereby increasing autophagy in A549 cells (Fig. 1a-f). These findings suggested that miR-193a controlled autophagy by regulating the p-Akt, Beclin1 and LC3-II pro-autophagy pathways.

### MiR-193a overexpression promoted autophagy and inhibited PQ-induced pulmonary fibrosis

**Fig. 2:**
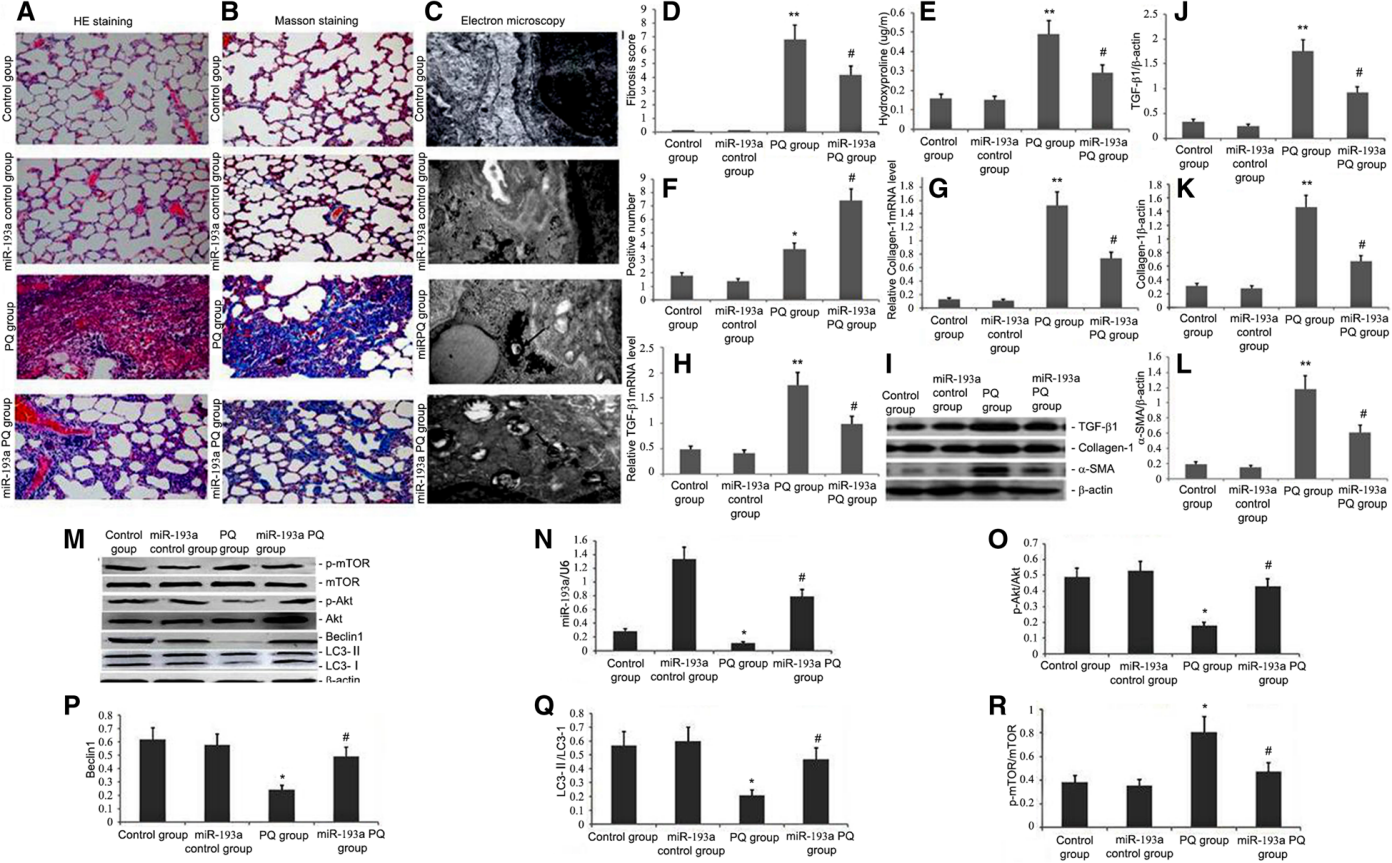
Overexpressing miR-193a effects on PQ-induced autophagy and pulmonary fibrosis. An active miR-193a expression construct was transferred to the mice by the intravenous tail administration of 1 × 10^9^ pfu AdCMV-miR-193a at 14 d after intraperitoneal PQ administration. **a**, **b** Representative images of Masson’s trichrome and H&E stained lung sections from the three experimental groups (magnification: 400×). **c** Autophagosome formation was detected in lung tissues (magnification: 15,000×). **d-e** Statistical analysis of the pulmonary fibrosis score and levels of hydroxyproline. **f** Six fields were selected randomly in each slice of lung and autophagosomes were calculated as the average positive rate. (Original magnification, 15,000×). **g**, **h** Representative statistical analysis of TGF-β1and Collagen I mRNA by qRT-PCR. **i**, **j-l** Representative graphs and statistical analyses of TGF-β1, α-SMA and Collagen I levels by western blot. **n** Representative statistical analysis of miR-193a by qRT-PCR. **m**, **o-r** Representative graphs and statistical analyses of Akt, mTOR, p-Akt, p-mTOR, Beclin1 and LC3-II levels by western blot. Data are expressed as the mean ± SD of three replicates. ^#^*p* < 0.05 vs. the model group; ^*^p < 0.05 vs. controls

To analyse the impact of overexpressing miR-193a on PQ-induced autophagy and pulmonary fibrosis, an active miR-193a expression construct was administered to mice via the intravenous tail administration of 1 × 10^9^ pfu AdCMV-miR-193a 14 d after the intraperitoneal administration of PQ. Administering AdCMV-miR-193a elevated autophagy in PQ-induced lung tissues and reduced PQ-induced pulmonary fibrosis (Fig. 2a-c). Histopathological evaluation of the lungs from PQ-treated miR-193a-overexpressing mice showed a marked decrease in collagen deposition and inflammatory cell infiltration compared with control animals (Fig. 2a-b).

### Effects of overexpressing miR-193a on autophagy protein p-mTOR, p-Akt, LC3-II and Beclin1 and profibrotic proteins TGF-β1, α-SMA and collagen I and genes TGF-β1 and collagen I mRNA

To explore the effects of overexpressing miR-193a on autophagy protein p-mTOR, p-Akt, LC3-II and Beclin1 and profibrotic proteins TGF-β1, α-SMA and Collagen I and genes TGF-β1 and Collagen I mRNA, an active miR-193a expression construct was administered to the mice by the intravenous tail administration of 1 × 10^9^ pfu AdCMV-miR-193a 14 d after the administration of intraperitoneal PQ. MiR-193a, TGF-β1 and Collagen I mRNA expression was measured by qRT-PCR, and p-mTOR, p-Akt, Beclin1, LC3-II, TGF-β1, α-SMA and Collagen I protein levels were measured by western blot. Administering AdCMV-miR-193a increased miR-193a levels in lung tissues, decreased p-mTOR levels, increased p-Akt, Beclin1 and LC3 levels (Fig. 2m, o-r), and decreased profibrotic proteins TGF-β1, α-SMA and Collagen I and genes TGF-β1 and Collagen I mRNA levels(Fig. 2h-l).

### Ligustrazin upregulated miR-193a expression

**Fig. 3:**
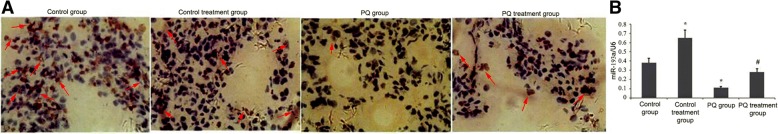
Effect of ligustrazin on miR-193a expression levels during PQ-induced pulmonary fibrosis. Mice were treated with ligustrazin and PQ for 14 d. miR-193a expression levels were determined with ISH. **a** ISH images from the four treatment groups, indicated with arrows (magnification: 400×). **b** Relative miR-193a expression levels were determined with qRT-PCR; bars indicate the mean ± SD. ^*^p < 0.05, ^**^p < 0.01 vs. controls; ^#^*p* < 0.05 vs. the model group

We then explored the effects of ligustrazin on miR-193a expression levels in fibrotic lung tissues obtained from mice administered ligustrazin and PQ for 14 d. MiR-193a expression levels in pulmonary tissues were measured with qRT-PCR and ISH. MiR-193a expression levels were lowered in the lungs during PQ-induced pulmonary fibrosis (Fig. 3). Conversely, miR-193a expression was significantly increased in ligustrazin-treated mice. ISH showed miR-193a expression in the cytoplasm of macrophages, lung alveolar epithelial type II cells, hyperplastic bronchiolar epithelial cells, and fibroblasts.

### Effect of ligustrazin on Akt, mTOR, LC3-II and Beclin1 levels in lung tissue

**Fig. 4:**
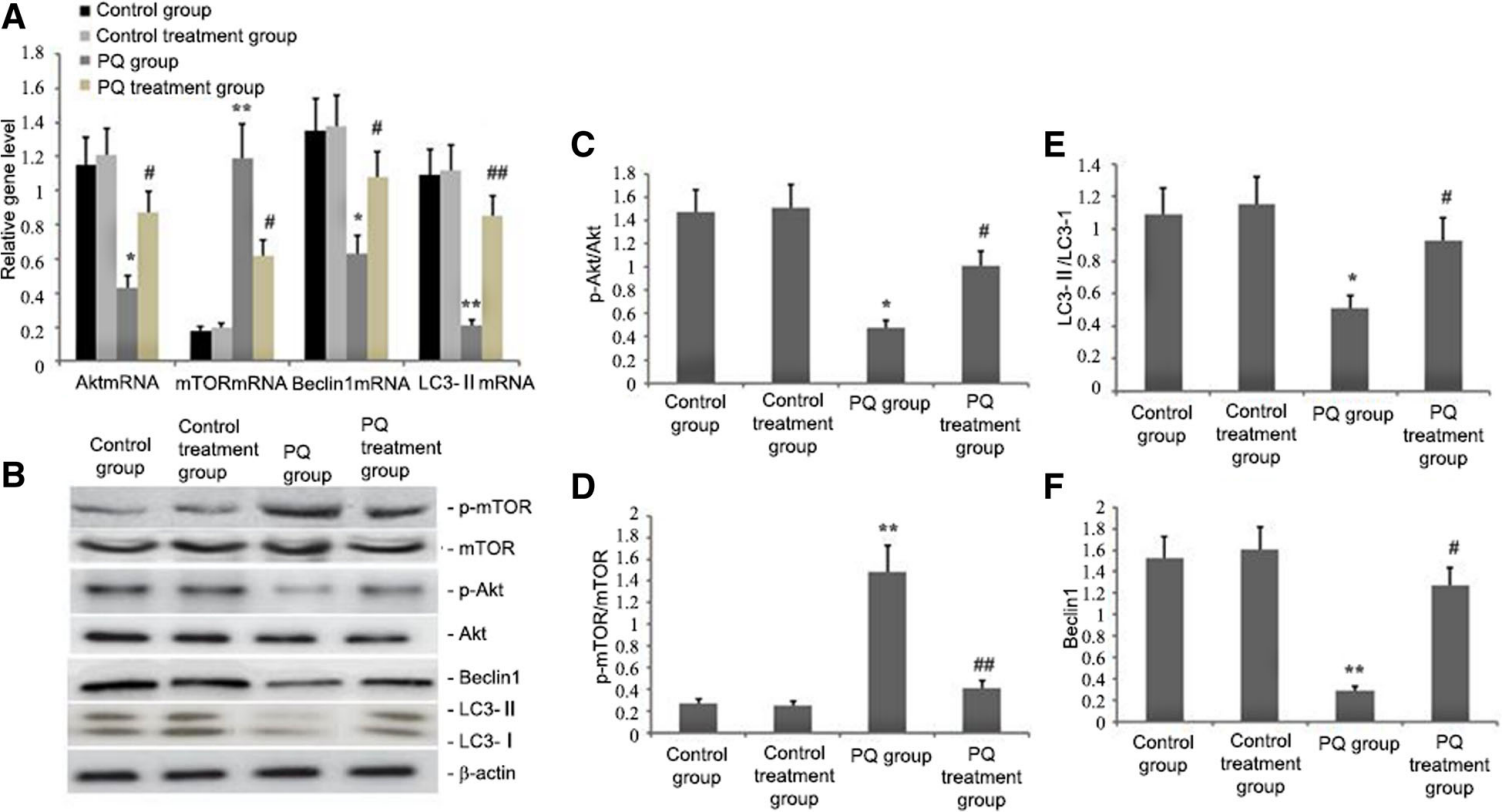
Effect of ligustrazin on Akt, Beclin1mTOR, and LC3-II mRNA expression levels during PQ-induced pulmonary fibrosis. Mice were treated with ligustrazin and PQ for 14 d. **a** Relative mRNA levels of mTOR, Akt, LC3-II, and Beclin1 were determined with qRT-PCR. **b-f** Relative levels of p-Akt, LC3-II, p-mTOR, and Beclin1 proteins were determined by western blotting. Bars indicate the mean ± SD. ^**^p < 0.01, ^*^p < 0.05 vs. controls; ^#^*p* < 0.05 vs. the model group

To evaluate the impact of ligustrazin on the expression of autophagy-associated genes in PQ-induced fibrotic lungs, we examined mTOR, Beclin1 and LC3-II mRNA levels in lung tissues of mice by qRT-PCR 14 d after ligustrazin and PQ treatment. mTOR mRNA levels in the lungs were elevated with PQ-induced pulmonary fibrosis. Beclin1, Akt, and LC3-II mRNA levels were decreased (Fig. 4a). In ligustrazin-treated PQ-induced mice, mTOR expression was significantly lowered and Beclin1, Akt, and LC3-II mRNA levels were increased (Fig. 4a).

### Effect of ligustrazin on p-Akt, p-mTOR, LC3-II and Beclin1 protein levels in lung tissue

The animals were treated with ligustrazin and PQ for 14 d, and then total protein from lung tissues was extracted and analysed by western blot. p-mTOR levels were significantly increased and LC3-II, p-Akt, and Beclin1 levels were significantly decreased in the PQ-induced fibrosis group in contrast to the control group (*p* < 0.01; Fig. 4b-f). In ligustrazin-treated PQ-induced mice, however, p-mTOR levels were significantly decreased and Beclin1, p-Akt, and LC3-II levels were increased in lung tissues.

### Effect of ligustrazin on autophagy throughout PQ-induced lung fibrosis

**Fig. 5:**
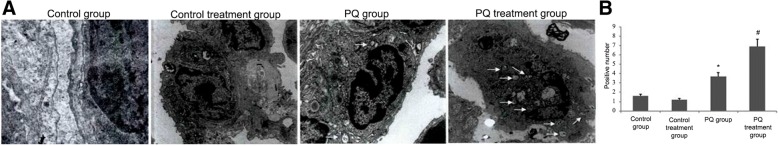
Autophagosome formation in lung tissues. **a-b** Autophagosomes in representative images from transmission electron microscopy (magnification: 15,000×, from the three treatment groups) are indicated with arrows. Six fields were selected randomly from each slice, and the average positive rate was calculated. Data are expressed as the mean ± SD of three experiments. ^#^*p* < 0.05 vs. the model group; ^**^p < 0.01 vs. controls

The animals were administered ligustrazin and PQ for 14 d, and then electron microscopy was used to evaluate the ultrastructures within cells to analyse the effect of ligustrazin on autophagy during PQ-induced lung fibrosis. Numbers of phagophores, autophagosomes and autolysosomes decreased in the PQ-induced pulmonary fibrosis group, but increased following ligustrazin treatment (Fig. 5). These results indicated that ligustrazin increased lung autophagy during PQ-induced lung fibrosis.

### Ligustrazin reduced SHH, Smo and Gli1 mRNA and protein levels in lung tissue

**Fig. 6:**
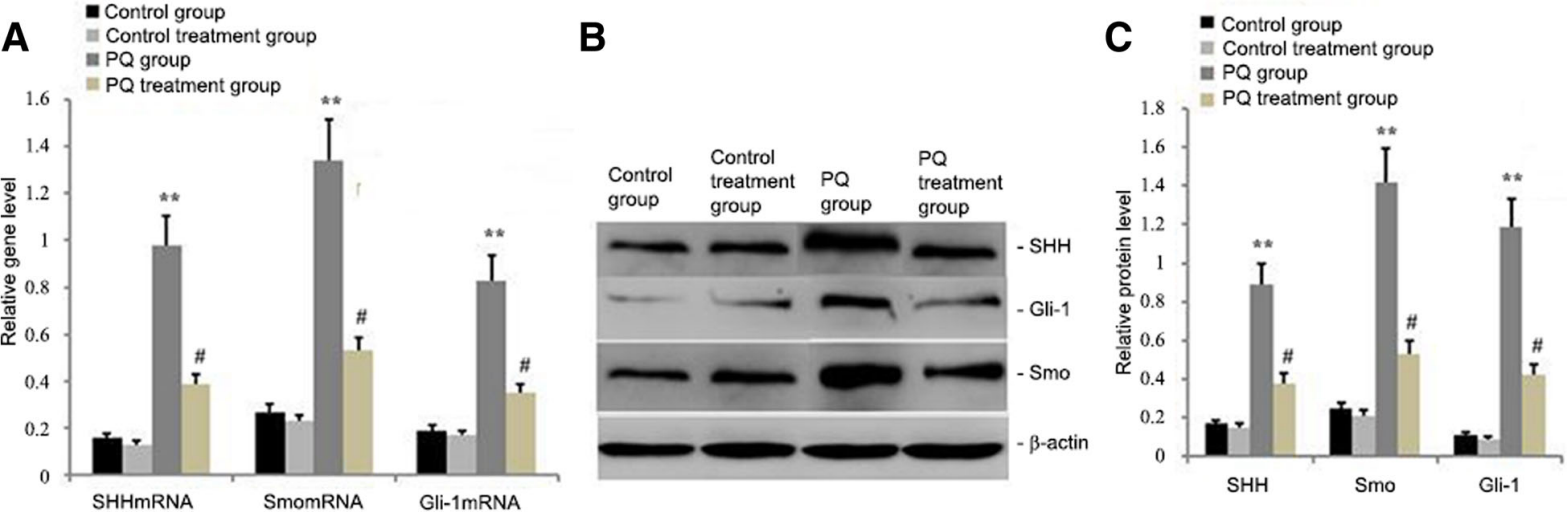
Effect of ligustrazin on Smo, SHH, and Gli1 mRNA and protein levels in murine lung tissues. Mice were treated with ligustrazin and PQ for 14 d. The relative levels of Gli-1, SHH, and Smo mRNA and protein expression levels were measured with qRT-PCR and western blotting, respectively. **a** Relative levels of Gli-1, SHH, and Smo mRNA. **b-c** Representative western blot results for Smo, SHH, and Gli-1 protein levels. All expression data were normalized to β-actin. Bars indicate the mean ± SD (*n* = 3). ^**^p < 0.01, ^*^p < 0.05 vs. controls

The animals were administered ligustrazin and PQ for 14 d. To analyse the effects of ligustrazin on PQ-induced lung fibrosis, Smo, SHH, and Gli1 mRNA and protein levels were identified in the lung tissues of the animals by qRT-PCR and western blot, respectively. During PQ-induced pulmonary fibrosis, Smo, SHH, and Gli1 levels increased in lung tissues (Fig. 6). In the PQ-induced mice treated with ligustrazin, Smo, SHH, and Gli1 expression were dramatically decreased.

### SHH is a direct miR-193a target

**Fig. 7:**
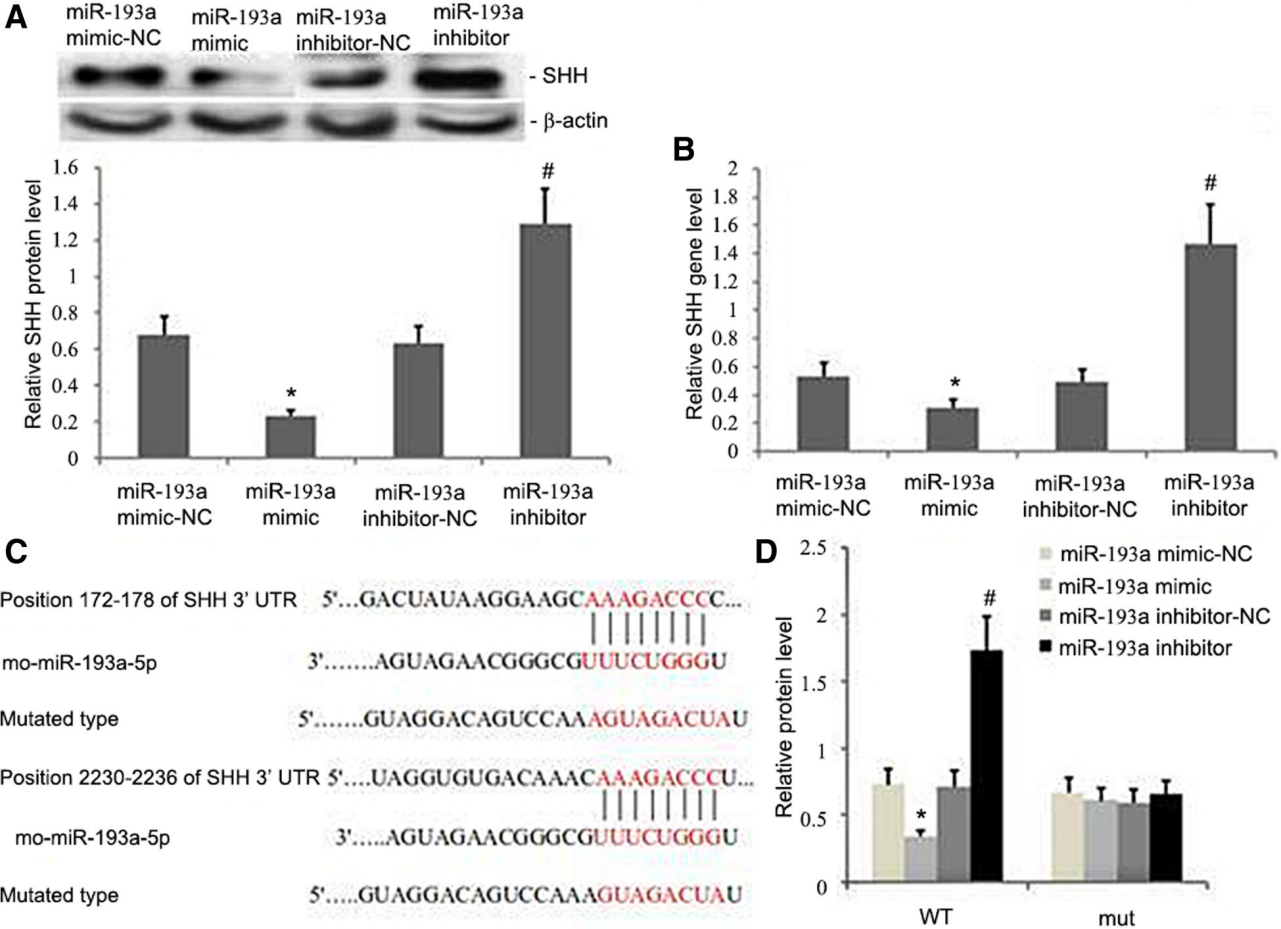
SHH is a miR-193a target. **a** SHH protein levels were reduced in miR-193a-overexpressing cells compared with vector controls (*p* < 0.05). **b** SHH mRNA levels were reduced in miR-193a-overexpressing A549 cells (*p* < 0.05). **c** miR-193a bound to SHH-3′UTR-wt. Binding was blocked by SHH-3′UTR-mt. **d** Dual Luciferase Reporter assays indicated that the miR-193a mimic bound SHH-3′UTR-wt but not the ones that were mutated (*p* < 0.05)

Using well-known databases to predict miRNA targets (see Methods), we found that SHH, an important profibrotic regulator of pulmonary fibrosis, was a candidate miR-193a target. SHH mRNA and protein levels were drastically lowered with overexpression of miR-193a (Fig. 7a, b). To further confirm the interaction between SHH and miR-193a, we constructed SHH-3′UTR-wt and SHH-3′UTR-mt constructs for Dual Luciferase Reporter Assays (Fig. 7c, d). As expected, miR-193a bound SHH-3′UTR-wt, but not the mutant version (Fig. 7d).

### Ligustrazin ameliorated oxidative stress

**Fig. 8:**
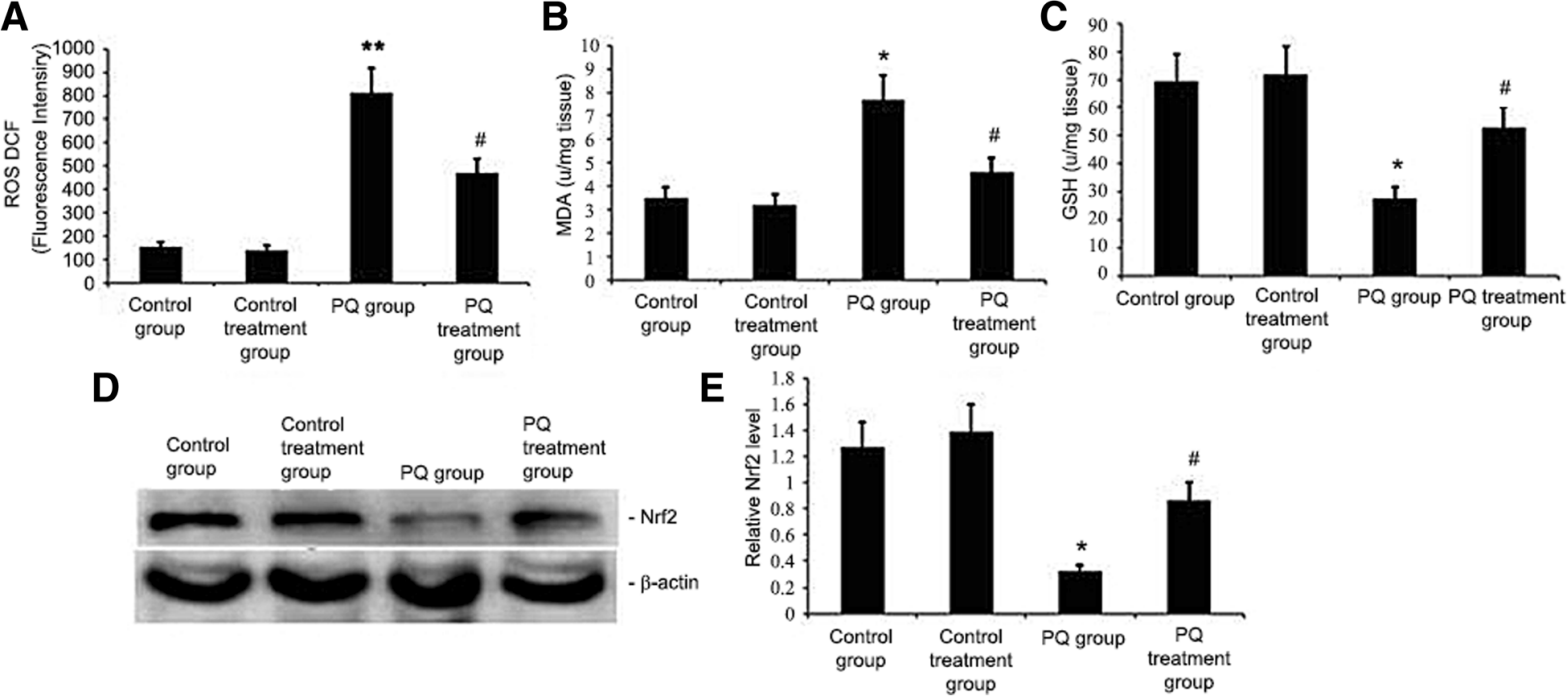
Effect of ligustrazin on ROS, Nrf2, MDA and GSH levels in lung tissues. Mice were treated with ligustrazin and PQ for 14 d. ROS,Nrf2, MDA and GSH levels were measured. The data are expressed as the mean ± SD, determined from three independent experiments. ^#^*p* < 0.05 vs. the model group; ^*^p < 0.05, ^**^p < 0.01 vs. controls

The animals were administered ligustrazin and PQ for 14 d. ROS, Nrf2, MDA and GSH levels were evaluated to assess the impacts of ligustrazin on oxidative stress. ROS and MDA levels were significantly elevated after 14 d of PQ treatment, however, GSH and Nrf2 were dramatically decreased (Fig. 8). However, ligustrazin significantly attenuated MDA and ROS expression and Nrf2 and GSH levels in PQ induced mice (Fig. 8).

### Ligustrazin reduced p-Smad2, CTGF, TGF-β1, α-SMA, collagen I, and collagen III levels in lung tissue

**Fig. 9:**
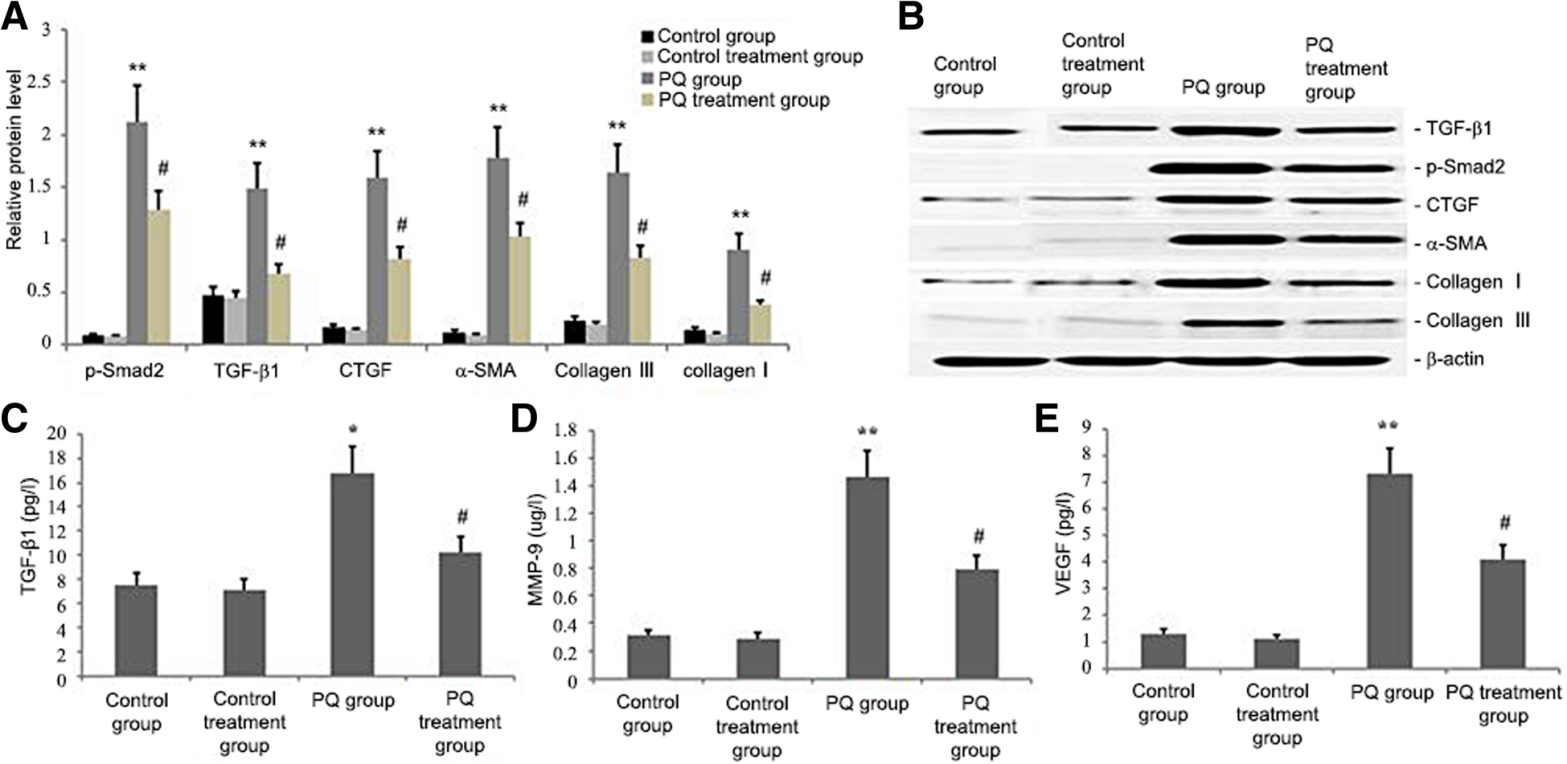
Effect of ligustrazin on p-Smad2, TGF-β1, CTGF, Collagen I, and Collagen III levels in lung tissues. **a**, **b** Representative western blots and statistical analyses of the densitometric data for TGF-β1, CTGF, Collagen I and Collagen III in mice at 14 d after ligustrazin administration. **c-e** Levels of TGF-β1, MMP-9, and VEGF in bronchoalveolar lavage fluids. Each value represents the mean ± SD of three independent experiments. ^##^*p* < 0.01, ^#^*p* < 0.05 vs. the model group; ^**^p < 0.01, ^*^p < 0.05 vs. controls

Mice were given ligustrazin and PQ for 14 d. TGF-β1, p-Smad2, Collagen I, and Collagen III protein levels during PQ treatment were quantified by western blotting to clarify the impact of ligustrazin on CTGF. TGF-β1, CTGF, p-Smad2, Collagen I and Collagen III protein levels in the lung were significantly elevated by PQ-induced pulmonary fibrosis in vivo (*p* < 0.05; Fig. 8). However, levels of all proteins were significantly lowered as an effect of ligustrazin (*p* < 0.05; Fig. 9).

### Impact of ligustrazin on α-SMA and Beclin1 localization in lung tissue during PQ-induced pulmonary fibrosis

**Fig. 10:**
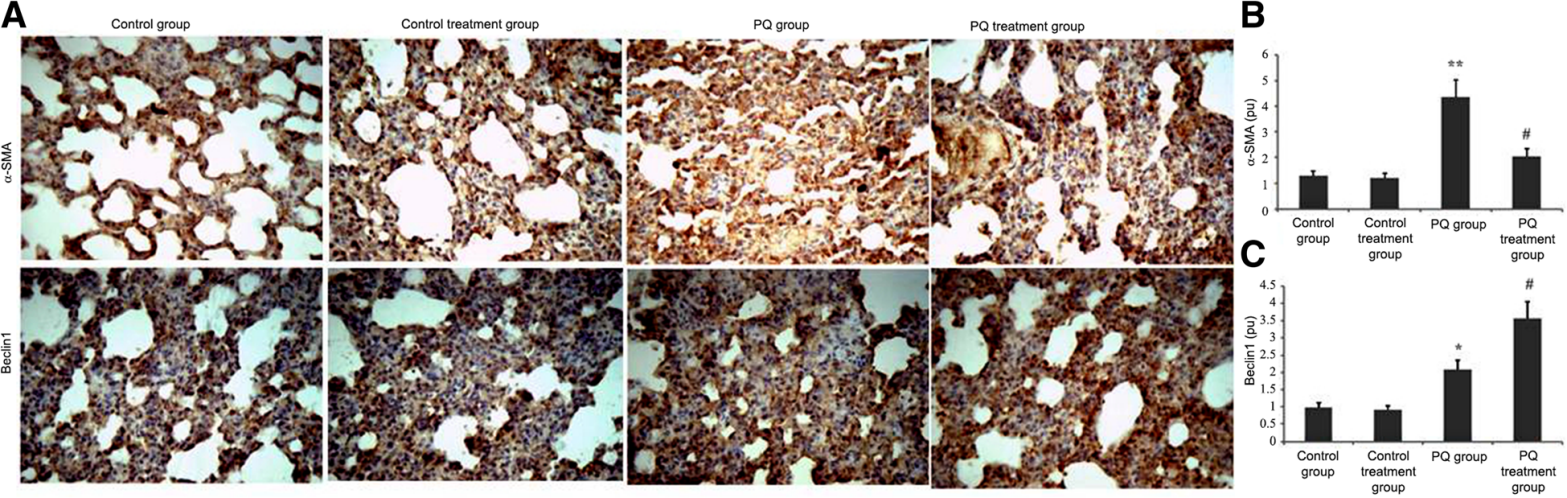
Effect of ligustrazin on α-SMA and Beclin1 protein expression levels in lung tissues during PQ-induced pulmonary fibrosis. Immunostaining was carried out on lung sections after antigen retrieval. **a** Representative immunostaining results, which showed α-SMA- and Beclin1-positive cells in the three treatment groups. **b-c** Statistical analyses of the densitometry data for α-SMA-positive and Beclin-positive cells in the three groups. All values are expressed as the mean ± SD. ^#^*p* < 0.05 vs. the model group; ^**^p < 0.01 vs. controls

Immunohistochemistry was conducted to evaluate the distribution of α-SMA and Beclin1 in the lung tissues at 14 d after PQ treatment. Positively immunostained cells were brown, and α-SMA and Beclin1 expressions were localized to the alveolar epithelium. The amount of positive cells for α-SMA and Beclin1 staining was significantly elevated in PQ-induced pulmonary fibrotic tissue. The rise in α-SMA was significantly lowered with ligustrazine. However, the increase in Beclin1 was further enhanced by ligustrazin treatment (Fig. 10a-h).

### Ligustrazin lowered TGF-β1, VEGF, and MMP-9 levels in bronchoalveolar lavage fluids from PQ-treated mice

The animals were administered ligustrazin and PQ for 14 d. Then bronchoalveolar lavage fluids were collected to measure TGF-β1, VEGF and MMP-9 levels. PQ induced significant pulmonary fibrosis, as demonstrated by the elevated concentrations of VEGF, TGF-β1, and MMP-9 in the bronchoalveolar lavage fluids. Ligustrazin reduced the increased secretion of MMP-9, VEGF, and TGF-β1 in PQ induced mice (Fig. 9d-f).

### Ligustrazin attenuated PQ-induced pulmonary fibrosis

**Fig. 11:**
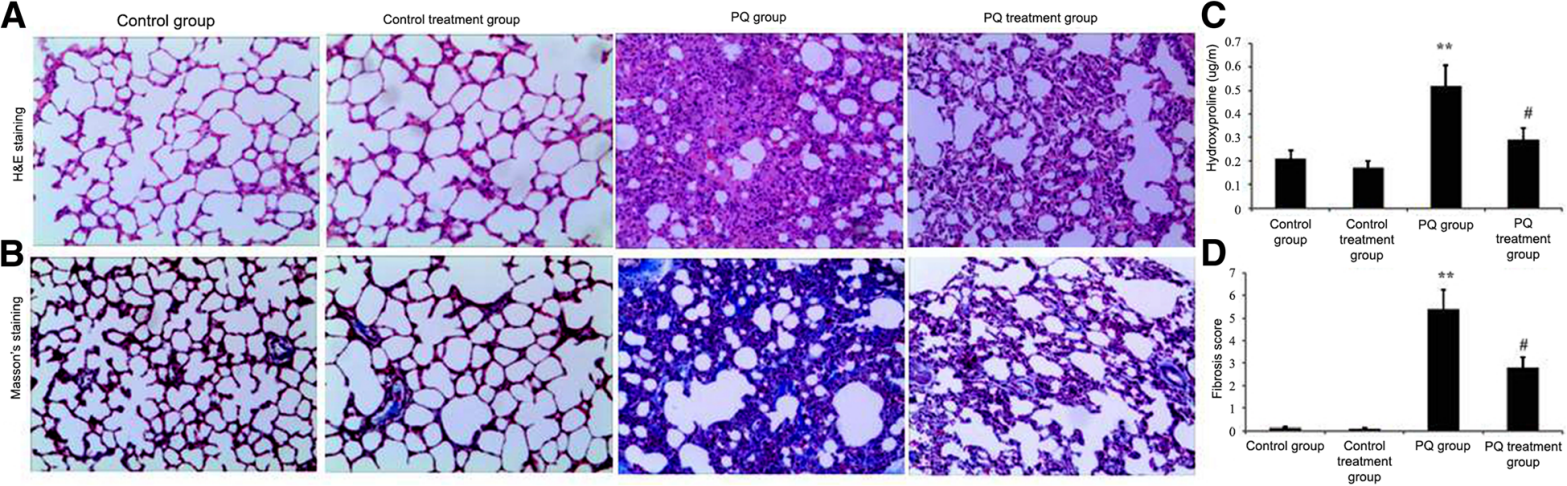
Histopathological analysis in lung tissues. **a-b** Histopathological changes in lung tissues in the different treatment groups. **a** H&E staining (magnification: 200×); (**b**) Masson’s trichrome staining (magnification: 200×). **c-d** Statistical analysis of the levels of hydroxyproline and pulmonary fibrosis scores. Pulmonary fibrosis scores are expressed as the mean ± SD of experimental results, performed in triplicate. #*p* < 0.05 vs. the model group; **p < 0.01 vs. controls

Finally, we evaluated the effect of ligustrazin on PQ-induced pulmonary fibrosis. H&E and Masson’s trichrome staining demonstrated a significant thickening of the alveolar septa and increased deposition of collagen in lung tissues at 14 d following PQ administration (Fig. 11a, b). The degree of pulmonary fibrosis was significantly mitigated after 14 d of ligustrazin treatment compared with PQ-treated mice.

Dense fibrosis with notable collagen deposition was seen in the lung tissues at 14 d following PQ administration. This dense fibrosis with collagen deposition was significantly ameliorated at 14 d after ligustrazin treatment compared with PQ-treated mice (Fig. 11a-c). No abnormal alveolar architecture was noted in the lungs of the control group. The degree of pulmonary fibrosis was evaluated with a scoring strategy that was described previously (see Methods). The scores of fibrotic lesions in PQ-treated mice were significantly elevated at 14 d after PQ administration in contrast to the controls (Fig. 11c). The scores were significantly lowered in ligustrazine-treated mice in contrast to PQ-treated mice. We additionally evaluated the level of pulmonary fibrosis by quantifying the HYP content in the lungs. The degree of pulmonary fibrosis was significantly increased at 14 d following PQ treatment (Fig. 11c). The HYP concentration, however, was significantly decreased in the ligustrazine-treated PQ-induced mice compared with the PQ-treated mice (Fig. 11c).

## Discussion

Pulmonary fibrosis is an important human health problem, but its precise pathogenic mechanisms remain unknown. PQ can cause toxicity by generating an oxidative stress response and fibrosis in a short time period, whereas pulmonary fibrosis has a slow and irreversible progression in patients. In this evaluation, the scores of fibrotic lesions were significantly elevated at 14 d after PQ administration. Masson’s trichrome and H&E staining showed significant thickening of the alveolar septa and collagen depositions (Fig. 11a-c). No interventions, however, have been effective in clinical studies on pulmonary fibrosis. The greatest promising intervention is ‘immunosuppressive’ therapy. This intervention, however, is not broadly utilized due to little supporting evidence [43, 44]. In this study, we showed that ligustrazin treatment significantly attenuated PQ-induced oxidative stress and also ameliorated PQ-induced pulmonary fibrosis (Fig. 11a-c).

PQ mainly accrues in the lungs via the polyamine uptake system [44, 45]. Excessive ROS may breakdown cellular macromolecules, which leads to tissue injury and fibrosis [46]. Nrf2 activation plays a pivotal part in antioxidant response element-driven expression of antioxidant and detoxifying enzymes, such as HO-1 [47, 48]. In our evaluation, ligustrazin elevated the expression of Nrf2, suppressed MDA and ROS activity, enhanced the activities of the antioxidants GSH and SOD (Fig. 8), which ultimately attenuated PQ-induced pulmonary fibrosis (Fig. 11c).

ROS initiate protective autophagy by regulating mTOR activity [49]. ROS disrupts the mitochondrial membrane potential in malignant gliomas and induces autophagy by inhibiting Akt/mTOR signalling [50]. Abnormal mTOR pathway activation is essential in fibrotic diseases [51] because mTOR signalling largely governs pulmonary fibrosis [52]. Several signalling pathways have been shown to be involved in regulating autophagy. Class-I PI3Ks (PI3K-I) inhibit autophagy by triggering mTOR [53]. ROS upregulates autophagy under inflammatory and oxidative stress conditions [44, 50]. As shown in Figs. 4, 5 and 11, the increase in ROS by PQ increased mTOR activity, inhibited autophagy and exacerbated PQ-induced pulmonary fibrosis. However, ligustrazin treatment significantly attenuated MDA and ROS levels, elevated Nrf2 and GSH expression, blocked mTOR activity, enhanced autophagy, and ameliorated PQ-induced pulmonary fibrosis.

Activated Akt (p-Akt) is a downstream effector of PI3K and it modulates the ‘double-edged sword’ function of autophagy [54]. The activation of TOR kinase is intermediated by PI3K, Akt and growth factor receptors [55]. Activation of the PI3K/Akt signalling pathway can restrain apoptosis and excessive autophagy and this can protect against pulmonary fibrosis [54]. In addition, Beclin1 and LC3-II are two significant markers of autophagosomes that are upregulated throughout reperfusion. They indicate chronic autophagy and cellular destruction. In this study, PQ treatment stimulated the PI3K/Akt signalling pathway and further increased mTOR activation, decreased Beclin1 and LC3-II expression, inhibited autophagy, and exacerbated PQ-induced pulmonary fibrosis (Figs. 4 and 5). However, PI3K/Akt signalling was inhibited by ligustrazin, which decreased mTOR activation, increased Beclin1 and LC3-II protein expression and autophagy, and ameliorated PQ-induced pulmonary fibrosis (Figs. 4 and 5).

Recently reported studies using animal models have suggested that Hh signalling has a role in fibrosis because it promoted myofibroblast differentiation [29]. Although Smo inhibitors suppress liver fibrosis [56], overexpressing SHH increases collagen expression [56]. Using an animal model of the liver, another current evaluation suggested the importance of Smo in α-SMA-expressing cells and fibrosis [57]. The Hh receptor Patched Homolog-1 (Ptch-1) prevented Hh signalling by inhibition of the co-receptor Smo [58]. Smo is released with binding of Hh ligands to Ptch-1 [28]. This results in the stabilization of Gli transcription factors, for example Gli1 [59], which stimulates target genes by binding to their Gli-binding consensus sequence (GACCACCCA) [60]. A previously reported study found that SHH induced myofibroblast differentiation of human lung fibroblasts in a Gli1- and Smo-dependent manner [56]. In this study, PQ inhibited the PI3K/Akt/mTOR autophagy signalling pathway (Fig. 4), enhanced Hh signalling by increasing ROS, upregulated Smo and Gli1 protein levels, promoted pro-Fibrin protein expression, and exacerbated PQ-induced lung fibrosis (Fig. 6). However, the PI3K/Akt/mTOR autophagy signalling pathway was blocked by ligustrazin, which increased autophagy, decreased Hh signalling (Figs.4, 5 and 6), downregulated Smo and Gli1 protein levels (Fig. 6), suppressed pro-Fibrin expression, and ameliorated PQ-induced lung fibrosis.

MiR-193 is involved in many important cellular processes [61, 62]. MiRNAs can halt the growth of lung cancer by inhibiting proteins that take part in autophagy [63]. MiR-193a inactivates the AKT/mTOR signalling pathway, and AMPK/mTORC1 serve as the centre of autophagy regulation [64]. Several miRNAs regulate AMPK/mTORC1. In our study, increasing miR-193a levels significantly increased p-Akt, Beclin1 and LC3-II levels, thereby increasing the level of autophagy in A549 cells (Fig. 1c and d). The administration of AdCMV-miR-193a increased miR-193a levels in lung tissue, decreased p-mTOR levels, increased p-Akt, Beclin1 and LC3 levels (Fig. 2m-r), increased autophagy levels in PQ-induced lung tissue, decreased profibrotic proteins TGF-β1, α-SMA and Collagen I and genes TGF-β1 and Collagen I mRNA levels(Fig. 2h-l), and reduced PQ-induced pulmonary fibrosis (Fig. 2a-b, d-e). These findings suggested that miR-193a could regulate autophagy and amend PQ-induced pulmonary fibrosis by regulating the Beclin1, p-Akt, and LC3-II pro-autophagy pathways. This study further showed that PQ-induced fibrosis reduced miR-193a activity (Fig. 3), significantly increased p-mTOR levels, decreased p-Akt levels, and significantly decreased Beclin1 and LC3-II protein expression (Fig. 4). However, miR-193a activity increased and p-mTOR levels were significantly decreased and p-Akt levels were significantly increased in ligustrazin-treated mice (Figs. 3 and 4). Furthermore, Beclin1 and LC3 levels were increased in lung tissue. The number of autophagosomes, phagophores, and autolysosomes were decreased in the PQ-induced pulmonary fibrosis group and they were increased in the ligustrazin treatment group (Fig. 5). Therefore, ligustrazin increased autophagy and improved PQ-induced pulmonary fibrosis by inactivating the AKT/mTOR signalling pathway by upregulating miR-193a.

Smad2 and − 3 are important in the TGF-β signalling pathway [46]. TGF-β1 is the main inducer of the production of the fibroblast extracellular matrix. It also promotes fibroblast to myofibroblast differentiation [51]. CTGF is the main downstream mediator of the activation of TGF-β-induced fibroblasts. Its particular effect on fibrotic tissues makes it a better therapeutic target than TGF-β [38]. Figures 3 and 9 show the attenuation of PQ-induced lung fibrosis by ligustrazin treatment and its association with increased miR-193a expression and blocking of the SMAD2/TGF-β1 signalling pathway and CTGF protein expression. This suggested that the alleviation of PQ-induced fibrosis might be assigned to the reduction of oxidative stress. Interestingly, CTGF was upregulated by VEGF [38], and inhibiting the VEGF pathway in pulmonary fibrosis might have protective effects against angiogenesis and fibrogenesis. According to our results, ligustrazin enhanced miR-193a expression, decreased VEGF and CTGF expression, suppressed ROS, and mitigated PQ-induced pulmonary fibrosis (Fig. 9).

α-SMA is a marker of fibroblast activation. Its expression provokes the transition of fibroblasts to myofibroblasts [28]. In this evaluation, significantly enhanced collagen deposition was seen in PQ-treated mice, which reflected the pivotal changes connected to fibrosis. Amplified HYP levels were shown to be correlated with collagen accumulation in the alveolar space. Our results suggested that ligustrazine-treated mice had significantly decreased α-SMA and HYP levels than mice in the model group (Fig. 9). The ameliorating impacts of ligustrazin on histological changes could be attributed to its radical scavenging activity. This activity prevented HYP accumulation in PQ-treated lung tissues. TGF-βl prompts a breakdown of collagen and other matrix proteins by improving the expression of MMP [38]. Collagen is the primary structural protein in animal bodies. In our study, ligustrazin treatment inhibited the expression of TGF-βl and MMP-9, suppressed total collagen, Collagen I and III, and attenuated PQ-induced pulmonary fibrosis.

## Conclusions

**Fig. 12:**
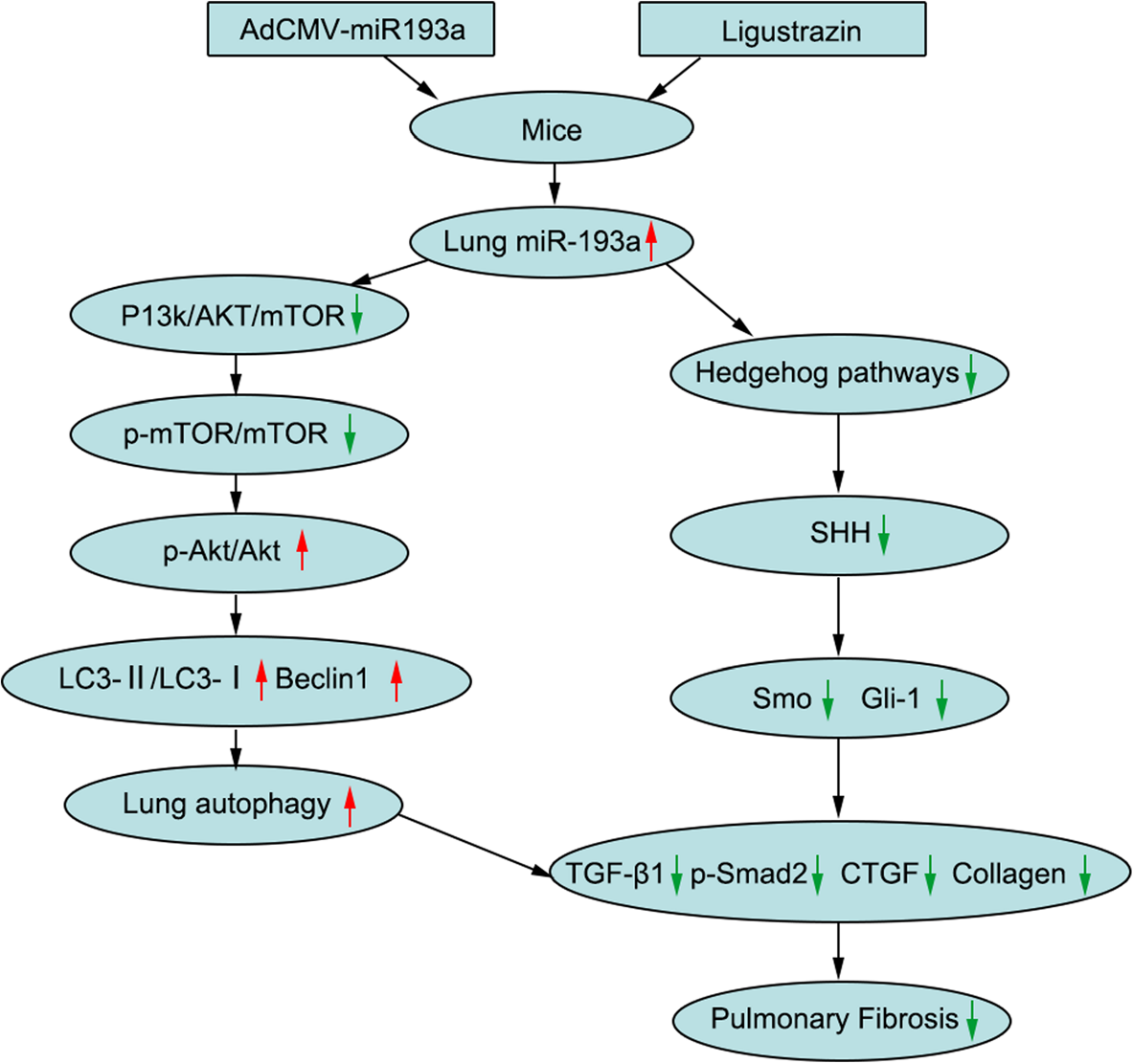
A potential unifying mechanism for the anti-fibrotic effects of ligustrazin

Although it has been reported that ligustrazin can attenuate pulmonary fibrosis in clinical practice, the associated molecular mechanisms have not been illuminated. This evaluation suggested a novel compensatory mechanism for ligustrazin in suppressing lung fibrosis under pathological oxidative stress conditions. Our study was the first to demonstrate that ligustrazin mediates anti-fibrotic protective effects in a model of PQ-induced pulmonary fibrosis. Outcomes revealed there was upregulation of miR-193a, a decrease in mTOR/Akt signalling, increase in Beclin1 and LC3-II expression, increased autophagy, attenuated Hh signalling, blocked Smo and Gli1 expression and inhibited pro-Fibrin expression (Fig. 12). The beneficial effects of the administration of ligustrazin in vivo on the parameters of pulmonary fibrosis might represent a new therapeutic modality for treating PQ-induced pulmonary fibrosis.

## Abbreviations

CTGF: Connective tissue growth factor
Hh: Hedgehog
HYP: Hydroxyproline
ISH: In situ hybridization
MDA: Malondialdehyde
miR-193: microRNA-193
mTOR: Mammalian target of rapamycin
Nrf2: Nuclear factor erythroid 2p45-related factor-2
P13K: Phosphoinositide 3-kinase
ROS: Reactive oxygen species
TGF-β: Transforming growth factor-β
VEGF: Vascular endothelial growth factor
α-SMA: α-smooth muscle actin
